# Halogen Migration in the Photofragmentation of Halothane

**DOI:** 10.3390/molecules30142902

**Published:** 2025-07-09

**Authors:** Anna Rita Casavola, Filippo Morini, Mattea Carmen Castrovilli, Jacopo Chiarinelli, Laura Carlini, Antonella Cartoni, Daniele Catone, Paola Bolognesi, Robert Richter, Bratislav Marinkovic, Sanja Tosic, Lorenzo Avaldi

**Affiliations:** 1 ISM-CNR-Istituto di Struttura della Materia, Area della Ricerca di Roma 1, CP 10, 00016 Monterotondo Scalo, Italy; matteacarmen.castrovilli@cnr.it (M.C.C.); jacopo.chiarinelli@cnr.it (J.C.); laura.carlini@ism.cnr.it (L.C.); antonella.cartoni@uniroma1.it (A.C.); paola.bolognesi@cnr.it (P.B.); lorenzo.avaldi@ism.cnr.it (L.A.); 2 Faculty of Sciences, UHasselt—Hasselt University, Agoralaan, 3590 Diepenbeek, Belgium; 3 Department of Chemistry, Sapienza University of Rome, P. le Aldo Moro 5, 00185 Rome, Italy; 4 ISM-CNR-Istituto di Struttura della Materia, Area della Ricerca di Roma 2, Via del Fosso del Cavaliere 100, 00133 Rome, Italy; daniele.catone@cnr.it; 5 Sincrotrone Trieste, Area Science Park, Basovizza, 34149 Trieste, Italy; robert.richter@elettra.eu; 6 Laboratory for Atomic Collision Processes, Institute of Physics Belgrade, University of Belgrade, Pregrevica 118, 11080 Belgrade, Serbia; bratislav.marinkovic@ipb.ac.rs (B.M.); seka@ipb.ac.rs (S.T.)

**Keywords:** halothane, halogen migration, photofragmentation, appearance energy

## Abstract

The photofragmentation of halothane (CF_3_CHBrCl) was studied with synchrotron radiation by photoionization efficiency (PIE) measurements and photoelectron–photoion coincidence (PEPICO) experiments, as well as by a theoretical exploration of potential energy surfaces. Among the other fragments, the formation of the CHClF^+^ and CHBrF^+^ ions, which involves the transfer of a F atom between the two moieties of the parent molecule, was observed. To understand the mechanisms leading to the halogen migration, a detailed theoretical study of the production of CHClF^+^, *m*/*z* 67^+^, based on DFT calculations and natural bond orbital (NBO) analysis was conducted. The results contribute to the understanding of the photochemistry of halothane, its polluting behavior in the high atmosphere, and the formation of highly reactive species.

## 1. Introduction

Recent works estimate that global health care contributes about 4–5% of the total greenhouse gas emissions. Among these, the direct emissions of anesthetics from patients can account for approximately 3% of the healthcare climate foot printing [1]. Establishing greenhouse gas inventories involves characterizing their physical and chemical properties, to understand their potential effects on atmosphere and climate.

**Scheme 1:**
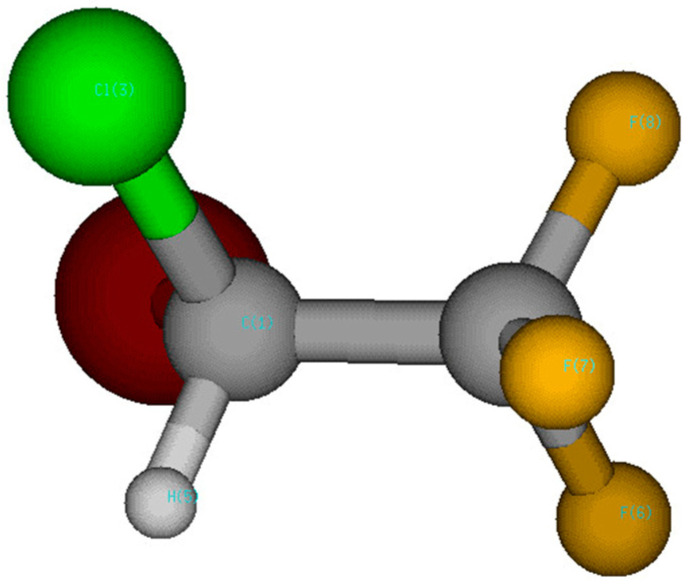
Halothane (CF_3_CHClBr) structure.

Among the fluorocarbons, halothane (CF_3_CHClBr), Scheme 1, containing three different halogen atoms, fluorine, chlorine, and bromine, is one of the most commonly used anesthetics. Despite the decrease in its use due to its hepatotoxicity, an estimate [2,3,4] of its worldwide use still amounts to several kilotons/yr. This has raised environmental concerns because those halogenated gases are emitted into the lower atmosphere, and Earth’s radiative balance may be disturbed by greenhouse heating that depends on their atmospheric lifetimes. Langbein et al. [4] calculated a value of ~100 yr for the photochemical lifetime of halothane at an altitude of 36 km. Halogenated gases, moreover, are a potential source of Cl and Br radicals that can contribute to stratospheric ozone depletion.

The knowledge of the photochemistry, photoionization, and photofragmentation of halothane can provide useful information for the studies of Earth’s radiative balance and ozone depletion.

There has been substantial interest in the photoionization and photodissociation of halogenated hydrocarbons regarding their variety of bond-breaking channels and due to the search for selective control of the relative yields of the ionization products. Ferreira da Silva et al. [5] recently reported an extensive characterization of the neutral excited electronic states and calculations of the ionization energies of halothane. In their paper they also review previous work on VUV absorption and photoemission. The EUV and soft X-ray-induced photofragmentation of the halothane molecule has also been investigated using time-of-flight mass spectrometry in the coincidence mode covering a broad energy region from valence (21.21 eV) to the carbon 1s edge [6] to search for site-selective effects. The same group also reported the ratios of double-to-total and triple-to-total photoionization cross-sections [7].

The positive and negative ion chemistry of halothane was also investigated by atmospheric pressure chemical ionization mass spectrometry (APCI-MS) in air plasma [8] and by means of collision-induced decomposition and ion/molecule self-reaction experiments [9]. Brown and co-workers [3] have measured the reactions of halothane with hydroxyl (OH) radicals, and Langbein et al. [4] measured the room temperature rate coefficient for reactions of the OH radical with halothane.

The two carbon atoms in halothane are embedded in completely different chemical environments. This has been exploited to distinguish halothane enantiomers (mirror isomers) and the determination of absolute configuration at a single-molecule level by four-body Coulomb explosion and to investigate the interplay of site-selective excitation and fragmentation patterns [10,11].

In this work, a combined experimental and theoretical effort has been made to investigate the VUV photofragmentation of halothane. The measured photoionization efficiency (PIE) curves and the photoelectron–photoion coincidence (PEPICO) yield have allowed the identification of several fragmentation channels and their appearance energy (AE). According to the established literature [12,13], the process is modeled considering the molecular electronic structure in its ground state, considering that the cation relaxes to its ground state with an available energy equal to the difference between the energy left in the molecule by the photon and the ionization potential. DFT calculations and natural bond orbital (NBO) analysis unveiled the mechanisms active along the ground state potential energy surfaces for the analyzed fragmentation paths. Particular attention has been devoted to the halogen transfer process, where a F atom from the CF_3_ moiety replaces one of the two halogens of the BrCHCl moiety.

## 2. Results

**Figure 1:**
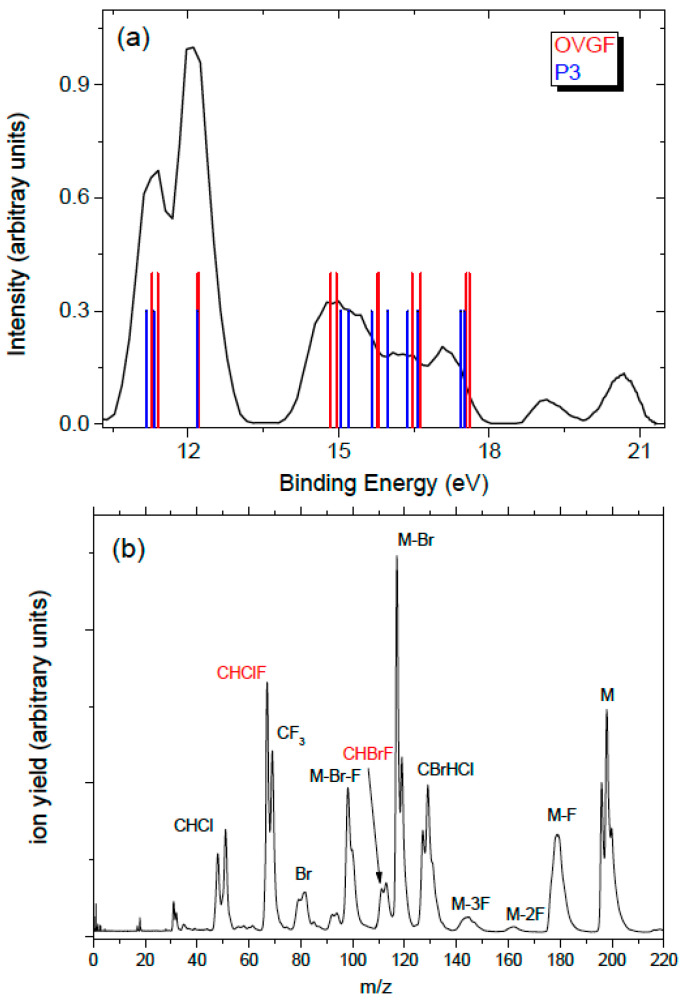
(**a**) The photoelectron spectrum of halothane at 21.5 eV. The bars are the calculated vertical ionization energies from ref. [5]; (**b**) the mass spectrum of halothane.

Halothane in the X ^1^A electronic ground state has a C1 symmetry. The calculated electron configuration of the outermost valence orbitals of the X ^1^A ground state is (41a)^2^(42a)^2^(43a)^2^(44a)^2^(45a)^2^(46a)^2^. The photoelectron spectrum of halothane (Figure 1a) displays features in three distinct regions. The highest occupied molecular orbital (HOMO) and the second highest occupied molecular orbital (HOMO-1) have a Br 4p lone pair character. The HOMO-2 (44a) has instead the Cl 3p lone pair character. The ionization potential of halothane determined by a high-resolution He I spectrum [14] is 11.2 eV. That spectrum showed the two Br states, 0.3 eV apart, which are not resolved in our spectrum (Figure 1a). The same occurs for the Cl 3p lone pair states, which are almost degenerated and have been measured at 12.2 and 12.3 eV [12]. The second region extends from about 14 to 18 eV with orbitals involving the C-Br and C-Cl bonds. On the high energy side of this region, the lowest energy orbitals involving the F atoms are also located. At higher binding energy a few broad bands are observed in the region extending from 18.5 and about 21 eV. In Figure 1a the predictions of the ionization energies using the P3 and OVGF methods from ref. [5] are also shown. The two predictions are very close to each other and reproduce the experimental results quite well.

In the mass spectrum (Figure 1b), the parent ion is characterized by a multiplet structure at *m*/*z* 196, 198, and 200, due to the combination of the Br (m = 79 and 81 Da with isotopic ratios about 1:1) and Cl (m = 35 and 37 Da with isotopic ratio 3:1) atoms; the peaks due to Br loss are located at *m*/*z* 117 and 119, and the peaks due to the additional nF (n = 1–3) losses are located at 117 and 119, 98 and 100, and 60 and 62, respectively. The breaking of the molecule at the C-C bond may produce either of the two moieties, CBrHCl^+^ (group at *m*/*z* 127/129/131) or CF_3_^+^ (*m*/*z* 69). Also clearly identifiable are the fragments CHClF^+^ (*m*/*z* 67 and 69) and CHBrF^+^ (*m*/*z* 111 and 113), which result from the migration of a F atom from the CF_3_ to the CBrHCl moiety and the loss of a Br or Cl atom. The peak at *m*/*z* 69 contains two contributions, one due to CF_3_^+^ and the other to CHClF^+^. With the present resolution in the mass spectrum, the group of peaks due to the Cl loss (m/z 161 and 163) overlaps with the one due to the 2F loss (*m*/*z* 158/160/162). Santos et al. [6] have reported the time-of-flight mass spectra of halothane at selected photon energies between 21.21 and 300 eV. All the fragments observed in Figure 1b are also present in the mass spectrum at 21.21 eV of [6], although in [6] not all the observed peaks have been assigned.

**Figure 2:**
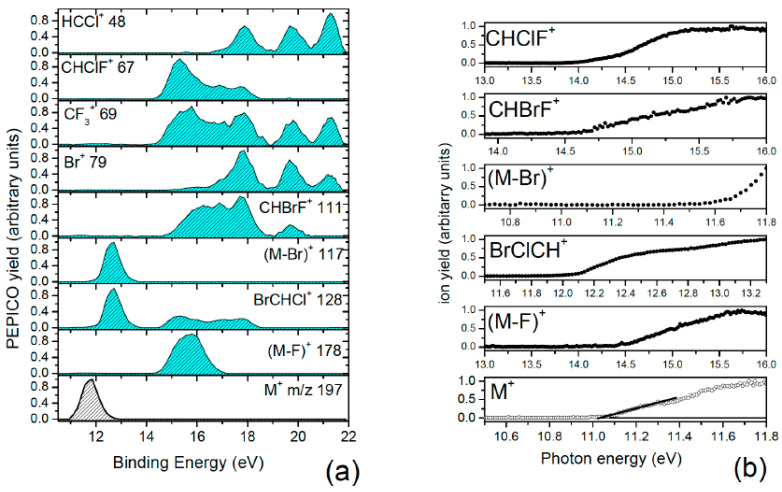
(**a**) The PEPICO yields of the fragments listed in the legend of the different panels; (**b**) the PIE curves of selected fragments used to calculate the AE. In the case of the parent ion, the fitting used to evaluate the AE_exp_ is shown.

In Figure 2a the relative PEPICO yields of the main fragments, independently normalized to 1, are shown. No attempt to calculate the branching ratio of the fragments has been made because the number of isotopes, in some cases overlapping, hampers a reliable evaluation of the contribution of different fragments under the same peak. In the analysis, only a specific isotope has been taken into account, as indicated in the legend of Figure 2a. In the case of the peak at *m*/*z* 69, in order to separate the contribution of the CF_3_^+^ ion from the one of the CH^37^ClF^+^, a third of the PEPICO yield of CH^35^ClF^+^ at *m*/*z* 67 has been subtracted, because the isotope ^35^Cl/^37^Cl ratio is 3. From the PEPICO yields, some information related to the AE and the relevance of the ionized orbitals in the formation of the fragment can be learned. More accurate values of the AE can be obtained by the PIE. The AE_exp_ values obtained by the PIE measurements in the present work are shown in Figure 2b and reported in Table 1, where they are compared with the theoretical predictions, obtained as described in Section 4. In the case of the PIE measurements, the procedure adopted in the case of the PEPICO yield to isolate the contribution of the CF_3_^+^ (*m*/*z* 69) cannot be carried out, because the PIE curves correspond to an independent scan for each fragment. Thus, the AE_exp_ in Table 1 is the one derived by the PEPICO experiments. The parent ion remains stable below 12 eV, and the first fragmentation channel to open is the Br loss, at AE ≈ 11.6 eV, which is consistent with the Br character of the HOMO and HOMO-1 orbitals. The F loss producing the CF_2_BrCHCl^+^ ion is observed in the second region of the PES spectrum, where contributions from orbitals involving the F atoms are expected, with AE_exp_ ≈ 14.22 eV. As far as the C-C bond breaking is concerned, the CF_3_ loss has an AE ≈ 12 eV while its complementary fragment, CF_3_^+^, appears at about 1.5 eV higher energy. From the measurements, it is observed that in the C-C bond breaking the charging of the BrCHCl moiety is favored up to about 14.5 eV; then both complementary fragments are observed up to about 18 eV. This may be due to the different structures of the ionic states formed in the photoionization process that may or may or not favor charge migration and, in turn, a given fragmentation pathway [15]. Above 18 eV the BrCHCl^+^ ion disappears, likely due to fragmentation. The further loss of the Br atom from this fragment leads to the HCCl^+^ fragment with AE ≈ 17 eV. The similar trend of this fragment and Br^+^ in the measured PEPICO yields suggests that BrCHCl^+^ fragmentation is the main channel to produce either Br^+^ or HCCl^+^ by the same bond break but alternative charge location. In Figure 2a the PEPICO yields for the production of the CHClF^+^ (*m*/*z* 67) and CHBrF^+^ (*m*/*z* 111) fragments are also reported. Both fragments are observed in region 14–18 eV, where orbitals involving the C-Br and C-Cl bonds are located. In a qualitative description these fragments result from a combined breaking of the molecule along the C-C bond, the loss of the Cl (or Br) atom, and the swapping of the Cl (or Br) atom with one of the F atoms of the other moiety. In the following section, we discuss in detail the fragmentation channel related to the C-C bond breaking leading to CHClF^+^ (*m*/*z* 67), providing evidence of the fluorine migration between these two moieties.

In Table 1 the AE_exp_ values are compared with the theoretical values obtained by the CCSD and CCSD(T) methods. Both methods are able to predict the whole of the expected fragments from the mass spectrum observed in the considered ionization regime. Despite the higher level in describing electron correlation of CCSD(T), the AEs predicted by this method are not significantly different from the ones predicted by CCSD. An overall good agreement between the experimental and theoretical values is observed in Table 1. The theoretical adiabatic ionization energy IE = 10.77 eV is in quite good agreement with the AE_exp_ = 11.05 ± 0.01 eV, while the vertical ionization energy predicted by the OVGF method, 11.127 eV, is in good agreement with the value (11.2 eV) of the high-resolution PES measurements [12]. The AE_th_ of CF_3_^+^ (12.97 eV) is lower than the experimental one, suggesting the existence of a transition state barrier that is not included in the calculations. In the case of the complementary moiety, CHClBr^+^, the calculated (11.93 eV) and experimental AE are very close.

## 3. Discussion: Halogen Migration

To understand the mechanisms leading to the halogen migration, a detailed analysis of the production of CHClF^+^, *m*/*z* 67, which considers the transition state energy barrier, was conducted.

**Figure 3:**
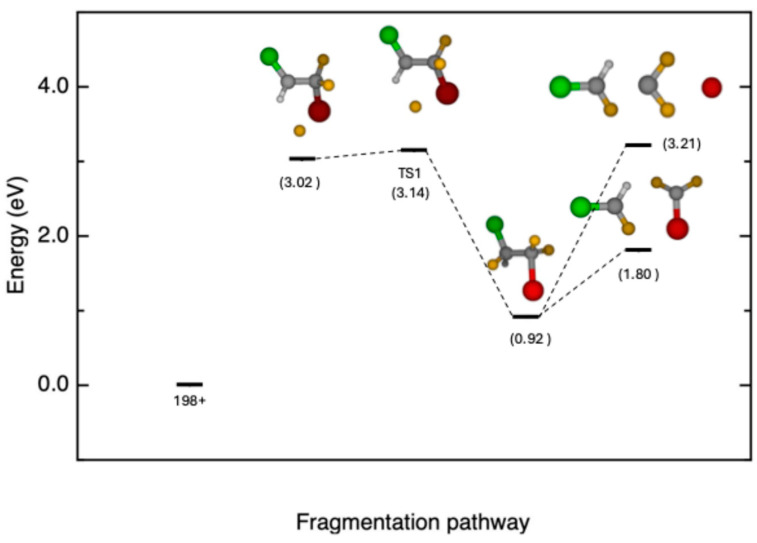
Schematic of the first reaction pathway, with inverted position between Br and F in halothane, which leads to the release fragment of CHClF^+^ (*m*/*z* 67, TS1 AE_th_ = 13.91 eV) and two distinct final states (CF_2_Br + CHClF^+^ 12.57 eV, CF_2_ + Br + CHClF^+^ 13.98 eV). Total relative energies (in eV with zero-point energy, ZPE) at the CCSD(T)/6-311++G** level of theory with respect to the parent ion.

The production of this fragment can be achieved via a complex path involving different steps. We focus our attention on two distinct reaction pathways. In the first one, the Br atom migrates to the adjacent C atom, resulting in a transition state where the F atom is positioned near the CHCl group (AE_th_ = 13.91 eV). Intrinsic reaction coordinate (IRC) calculations (see Supporting Information Figure S1) reveal that the product corresponds to a structure where the F and Br atoms are inverted (Figure 3). From this configuration, a potential energy surface scan was performed, leading to the *m*/*z* 67 fragment and yielding two distinct final states, depending on the configuration of the CF_2_Br group, which can either be bonded or exist as separate CF_2_ and Br entities (see Figure 3).

**Figure 4:**
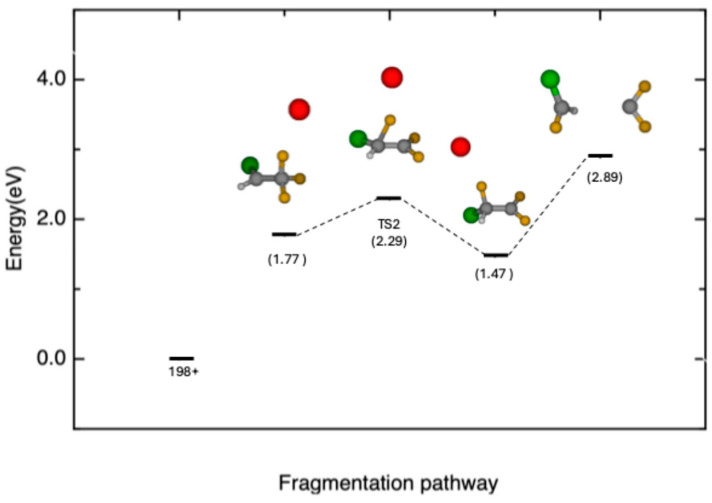
Schematic of the second reaction pathway, leading to the release of fragment CHClF^+^ (*m*/*z* 67, TS2 AE_th_ = 13.06 eV). Total relative energies (in eV with zero-point energy, ZPE) at the CCSD(T)/6-311++G** level of theory with respect to the parent ion.

In the second pathway, depicted in Figure 4, the Br atom is initially ejected; then a transition state in which the F atom migrates to the adjacent C atom (TS2 AE_th_ = 13.06 eV) is formed. The intrinsic reaction coordinate (IRC) calculations for this case are reported in Figure S2 of the Supporting Information. As in the first pathway, after the migration, the potential energy surface can be explored until the C-C bond breaks, leading to the formation of the *m*/*z* 67 fragment (13.66 eV).

As previously observed in the case of pyrimidine [16] and halogenated pyrimidines [17], there is a correlation between the molecular orbitals (MOs) and peculiar fragmentation patterns. The present OVGF and P3 calculations with the 6-311++G** basis set of the ionization energies of halothane are collected in Table 2.

**Figure 5:**
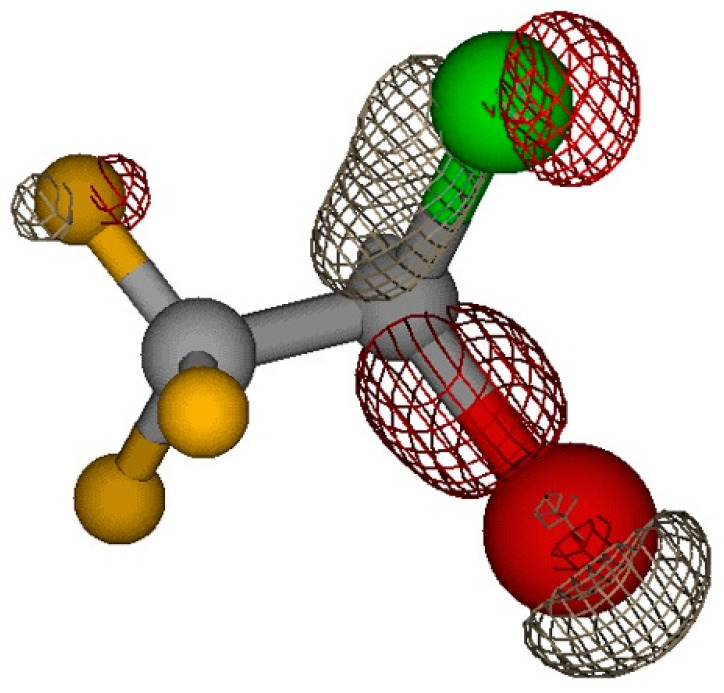
Electron density distribution of orbital 42a.

From the analysis of the electron density of the different molecular orbitals, the orbital 42a (OVGF ionization energy 14.7 eV) can possibly be involved in the fragmentation path leading to fragment *m*/*z* 67. The electron distribution of the orbital 42a, shown in Figure 5, is located along the C-Br and C-Cl bonds.

A detailed natural bond orbital (NBO) analysis of the structures of halothane (neutral, parent ion or cation, TS1, and TS2) was performed to understand the path leading to the halogen migration. The results are reported in Table 3, Table 4, Table 5 and Table 6. The notation of the orbitals, according to the standard output of the NBO code, is as follows: “BD” for 2-center bond, “LP” for 1-center valence lone pair, “RY*” for 1-center Rydberg, and “BD*” for 2-center antibond. In the case of the open-shell structures, the NBO analysis provides the separate contribution to the overall stabilization given by the alpha and beta spin orbitals. This is visible in Table 4, Table 5 and Table 6. Several studies have considered the structural characteristics of halothane [18,19,20,21,22]; however none of them provide a more in-depth use of natural orbitals, with the exceptions of [18,20], where limited information on donor–acceptor interactions and natural charges is available. Natural bond orbital analysis is a very good method for studying intra and intermolecular bonding and donor–acceptor interaction and may provide a strong insight into the presence of charge transfer or conjugative effects. The larger the value of the stabilization energy E(2), the stronger the donating tendency from electron donors to electron acceptors is expected to be, implying a more extensive conjugation for the whole system. NBO analysis has been then performed to elucidate the role of electron delocalization in the reactant and the stabilization of the transition state. In addition to that, NBO analysis has proven its ability to provide quantitative information on the study of transition states in the reaction mechanisms of organic molecules [23,24].

**Figure 6:**
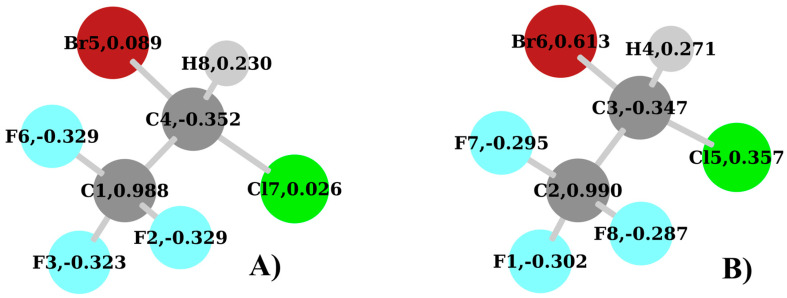
Natural charges of halothane at the B3LYP/aug-cc-pVTZ: (**A**) neutral and (**B**) cation.

**Figure 7:**
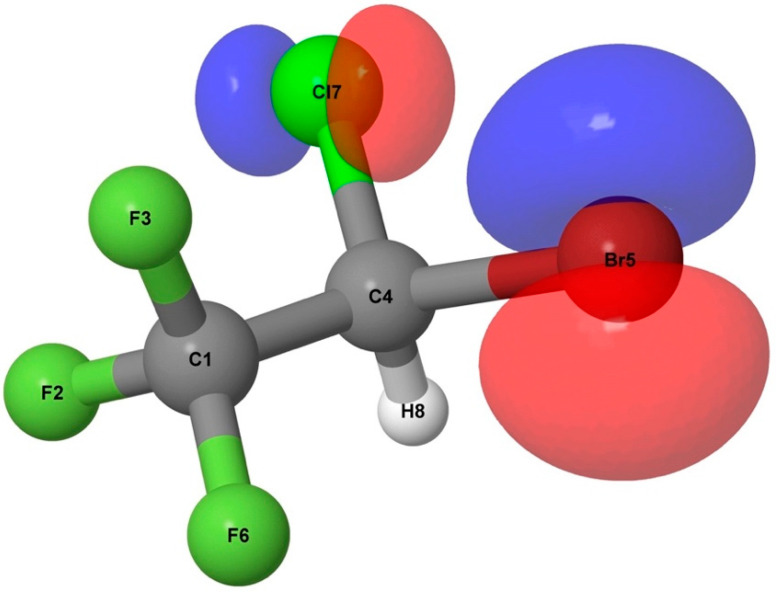
HOMO of halothane at the B3LYP/aug-cc-pVTZ level.

In Figure 6 the natural charges of the neutral halothane molecule and the parent ion are reported. The main difference here is related to the charge on the Cl and Br atoms. While on the neutral molecule these are almost neutral, in the parent ion a positive charge is localized on these atoms. This is due to the localization of the entire charge distribution of the HOMO orbital on the Cl and Br atoms (see Figure 7). Moreover, upon its formation, the cation undergoes a significant structural change with the Cl-C-Br angle varying from 112.4° to 95.1° in the neutral molecule and the cation, respectively. These differences may deeply affect the overall stability by limiting the adequate overlap among the natural orbitals within the donor–acceptor scheme. This alteration is clearly visible by comparing the number of interactions with E(2) ≥ 5 kcal/mol (see Table 3 and Table 4).

By looking at Table 3, we see that the neutral form of halothane is stabilized almost completely by the interaction due to the delocalization of the F atoms lone pairs (LP) on the orbitals involving the C atom directly bonded to them. This is clearly due to the positive charge (0.988) that is present on this C1 atom (Figure 6A). Most of these interactions have an E(2) > 10.0 kcal/mol. The same is not possible for the lone pairs of Cl and Br, because the C4 atom (Figure 5) has a negative charge (−0.352) while these two atoms are almost neutral. Only two interactions are observed in this sense in Table 3: the 40.LP(3)Br5 => 361.BD*(1)C4-Cl7 with E(2) = 6.40 kcal/mol and the 46.LP(3)Cl7 => 360.BD*(1)C4-Br5 with E(2) = 9.13 kcal/mol.

When the parent ion is considered, a significant drop in the number and strength of stabilization interactions is found with respect to the neutral molecule. The removal of one electron increases what can be defined as a “confinement effect” in terms of donor–acceptor interactions. Indeed, here the whole stabilization is related just to a very limited number of interactions involving the lone pairs of F atoms and the orbitals related to the C1 atom. It is interesting to notice that, despite the removal of an electron, no significant charge rearrangement is found for the carbon atoms, the fluorine atoms, and the hydrogen. Overall, the two groups, CF_3_ and CHClBr, at least in the neutral and in the cation, seem to have very little interaction with respect to each other and have the tendency to show a strong charge separation.

**Figure 8:**
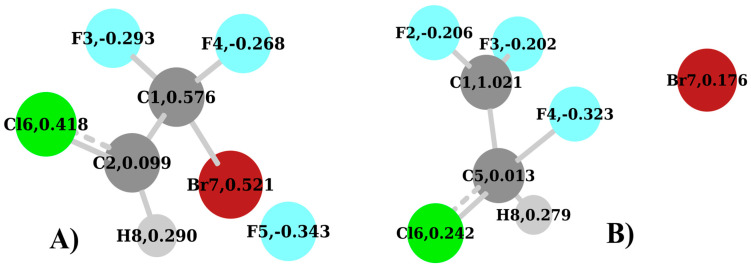
Natural charges of halothane at the B3LYP/aug-cc-pVTZ: (**A**) TS1 and (**B**) TS2 (see also Figure 3 and Figure 4).

The next structures to be considered are the TS1 and TS2. These, along with the calculated natural charges, are shown in Figure 8A,B, respectively. In the TS1 (Figure 8A), similarly to the parent ion, the Cl and Br atoms have a relatively strong positive charge of 0.418 and 0.521, respectively, while the C1 atom now bonds to the Br atom, due to the F loss, and has a charge of 0.576. This is a strong variation with respect to the parent ion where the corresponding carbon atom, C2 (Figure 6B), has a charge of 0.990. Here the positive charge is shared between the two atoms, C1 and Br. Moreover, the C2 atom in the TS1 (Figure 6B) is substantially neutral with a charge of 0.099. This shows that there is no relevant interaction with the detaching F5 atom.

When the TS1 is considered (see Table 5), very few donor–acceptor interactions have any relevance, as in the case of the parent ion. The strongest is 360.BD*(2)C2-Cl6 => 359.BD*(1)C2-Cl6 (beta spin) with E(2) = 11.59 kcal/mol. Despite the very low occupancy of these orbitals (0.077 and 0.048, respectively), this result is due to the small value of E(j)-E(i) = 0.10 and the large value of the representative off-diagonal element in the Fock matrix F(i,j) = 0.123. A similar consideration can be made for the interaction 361.BD*(2)C2-Cl6 => 360.BD*(1)C2-Cl6 (alpha spin) where the E(j)-E(i) = 0.12 and the F(i,j) = 0.118, where the respective occupancies of the orbitals are 0.068 and 0.038. Moreover, in the case of the TS1, the major stabilizing interactions are related to the F atoms acting as donors.

**Figure 9:**
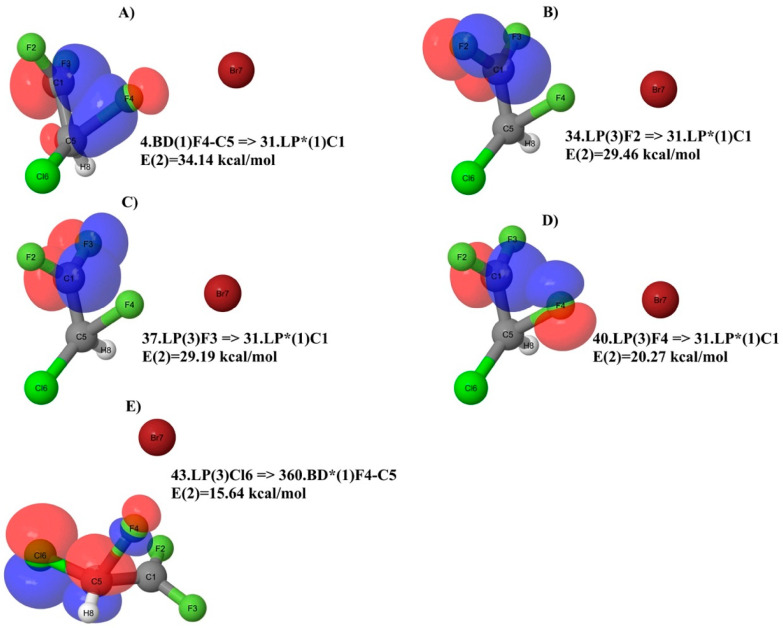
Major NBO donor–acceptor interactions of TS2 for alpha spin.

**Figure 10:**
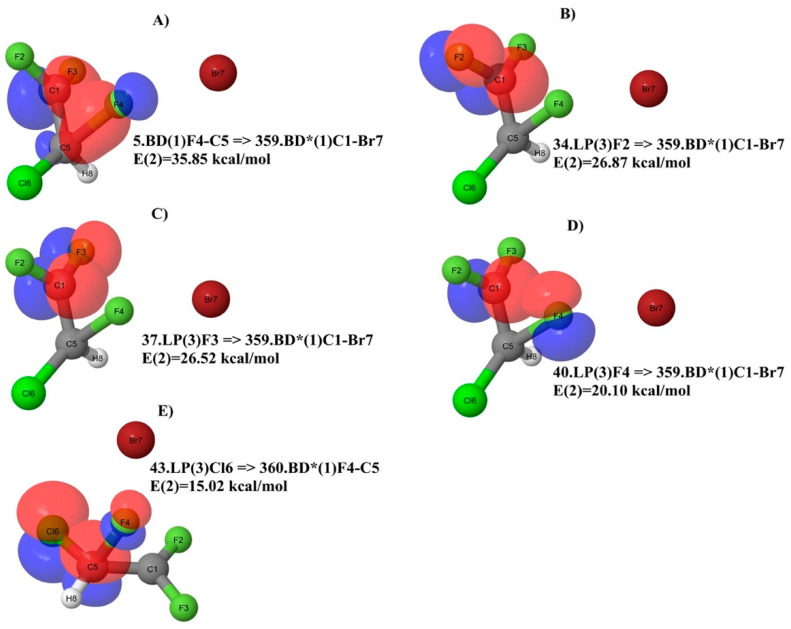
Major NBO donor–acceptor interactions of TS2 for beta spin.

When the TS2 (Figure 8B and Table 6) is analyzed, two major differences with respect to the TS1 must be taken into account. The first one is the migrating F4 atom that forms a bond with the C5 atom. The second one is the charge of 1.021 on the C1 atom in the CF_2_ group, which has lost the F4 atom. This is very similar to the one of the neutral molecule (Figure 6A) and parent ion (Figure 6B). In this regard, it is useful to consider the geometry of the TS2. We can see in Figure 8B that the migrating F4 atom is placed at 1.73 Å from the C5 atom, and at 1.83 Å from the C1 atom. Moreover, the angle F4-C5-C1 is 70.0°, while the angle F4-C1-C5 is 62.38°. This arrangement, in conjunction with the positive charge on the C1 atom of the CF_2_ group, is responsible for the two strongest donor–acceptor interactions, which can be visualized in Figure 9A and Figure 10A, respectively. The first one is 4.BD(1)F4-C5 => 31.LP*(1)C1 (alpha spin) with E(2) = 34.14 kcal/mol (Table 6). This involves the F4-C5 σ bond with the antibonding lone pair-type orbital located on the C1. The second donor–acceptor interaction is 5.BD(1)F4-C5 => 359.BD*(1)C1-Br7 (beta spin) with E(2) = 35.85 kcal/mol (Table 6). Beyond that, the new position of the F4 atom in Figure 8B accounts for the interactions in Figure 9D,E and Figure 10D,E as well. In general, at this level, if we look at Table 6 and Figure 9 and Figure 10, the NBO analysis accounts for strong conjugation effects, which in the TS2 are due to the F p lone pairs orbitals acting as the main donors and π* orbitals as the main acceptors in the stabilization of this carbocation. With very few exceptions, the antibonding orbitals involving the C1 atom of the CF2 group are those playing a major role as acceptors. If we look at the similarities in terms of NBO analysis among the species considered here, the TS2 (Figure 8B) shares some similarities with the neutral molecule (Figure 6A), because of the number of interactions involving F atoms as donors and the C1 atom directly attached to them as the acceptor. This may suggest a direct link between the two species when the structural rearrangement and fragmentation paths are considered, which might not involve the parent ion.

To clarify these issues, further investigation, which is beyond the scope of this paper, may be considered. The presence of hyperconjugation should be considered [25] in order to have an in-depth overview of the donor–acceptor interactions of halothane upon ionization and to further extend this investigation to all possible structural and fragmentation paths.

In summary, the NBO analysis suggests that the preferred fragmentation pathway leading to fragment CHClF^+^ (*m*/*z* 67) proceeds via TS2 rather than TS1, as TS2 exhibits a larger number of stabilization interactions typical of a carbocation. This is confirmed by the relative energy difference between the two species of TS, with the TS2 being 19.6 kcal/mol lower than the TS1.

## 4. Materials and Methods

### 4.1. Experimental

Two different types of experiments have been performed at the CiPo beamline [26] of the Elettra-Sincrotrone Trieste laboratory (Trieste, Italy): the photoionization efficiency (PIE) measurements to determine the appearance energy (AE) of the fragments and the photoelectron–photoion coincidence (PEPICO) measurements to measure the relative fragmentation yield at different photon energies. The radiation was produced by an electromagnetic elliptical undulator/wiggler [27] and monochromatized by using a normal incidence monochromator (NM) equipped with two different gratings. In the PIE measurements the aluminum grating was used. The setup consists of five electrostatic lenses that focus and accelerate the ions from the region of interaction to the quadrupole mass spectrometer (QMS). This is a commercial QMS (10–4000 u, Extrel (Pittsburgh, PA, USA) 150-QC 0.88 MHz) with a mass resolution ΔM/M of about 500. It is mounted perpendicular to the photon beam and to the gas source, which is an effusive beam. In these measurements the ion yield of the selected fragment was measured versus photon energy with a photon energy resolution of about 100 meV and a photon energy step size of 20 meV. The PIEs were normalized to the photon intensity, which was measured simultaneously by a photodiode located at the end of the beamline. The photon energy was calibrated against the auto-ionization features observed in the Ar total photoionization efficiency spectra between the 3p spin orbit components. In the photon energy scans up to 11.7 eV, a lithium fluoride filter was used to remove the second-order radiation. Above this energy, the contribution of the second-order radiation was evaluated by comparing the Ar^+^ ion yield measured as a function of the photon energy to its ionization cross-section [28]. This second-order contribution was taken into account in the extraction of the PIEs.

**Figure 11:**
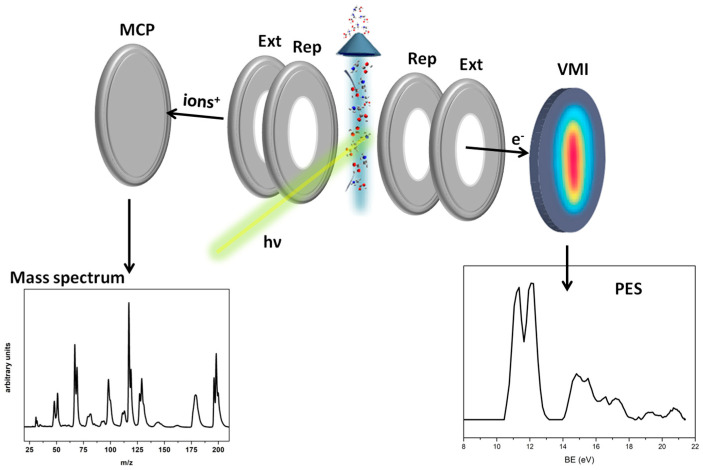
Scheme of the VMI-ToF spectrometer. In the two bottom panels, the photoelectron spectrum (PES), (**right**), and the mass spectrum, (**left**), of halothane measured on the VMI and TOF detectors, respectively, are shown (see Figure 1 for detailed plots of PES and MS spectra).

In the second type of experiments, the photoelectron spectra (PES), the mass spectra, and the PEPICO spectra were measured using a velocity map imaging (VMI) spectrometer. The end station (Figure 11) consists of a VMI spectrometer equipped with a position-sensitive crossed delay line anode and a time-of-flight (ToF) spectrometer with a microchannel plate detector, mounted opposite each other. The end station can be operated in two modes: ion on VMI and electrons recorded on the TOF detector or electrons on VMI, in order to acquire their kinetic energy and angular distributions, while the ions are directed towards the TOF detector. In the present experiments the second mode with electrons on VMI was used. The measurements were carried out in pulse-counting mode. The signal was amplified and discriminated and then transmitted to an 8-channel time-to-digital converter (THR08-TDC). This particular TDC was developed explicitly for conducting coincidence measurements using delay line detectors [29]. The PES, the mass spectra, and the PEPICO measurements were collected at hv = 21.5 eV using the Au grating of the NIM of the CiPo beamline. The velocity-to-energy conversion for the photoelectrons was calibrated using the photoelectron spectra of neon and xenon. The ΔE/E resolution of the imaging assembly is 6%. The velocity map images of the photoelectrons were then processed to obtain the PES spectrum (Figure 11).

Further details of the setups and the experimental procedures are described by Castrovilli et al. [30] for the PIE measurements and reported by O’Keeffe et al. [31] for the VMI setup and experiments.

Anhydrous halothane with a purity of +99% was degassed via freeze–pump–thaw cycles and then let into the chamber via a gas inlet. The pressure during the experiment was 6.2 × 10^−7^ mbar. The total acquisition time in the PEPICO experiments was about 6 h.

### 4.2. Theoretical

The AE is the minimum energy required to produce a particular fragment ion. Theoretically, the AE is defined as the difference between the energy of the ground state of the fragment and that of the neutral molecule (i.e., the adiabatic energy of the fragmentation process). In this work, we performed ab-initio calculations of the AE for the parent ion and some ionic fragments of halothane using the theoretical methods embedded in the Gaussian 16 package of programs [32]. The theoretical values, AE_th_, calculated here at 298.15 K, can be equal to or lower than their corresponding experimental ones (AE_exp_) as they do not take into account the transition states (TS, reverse activation barrier) for each fragmentation channel, kinetic shift, unfavorable Franck–Condon factor, or possible excited state which may affect the fragmentation processes. In all calculations, the fragmentation was considered to proceed from the ground state of the molecular ion.

The studied geometries have been optimized by means of density functional theory (DFT) along with the Becke 3-parameter LeeYang–Parr (B3LYP) function [33,34] and the 6-311++G** basis set. The chosen methods and basis sets are the results of a thorough preliminary investigation where different approaches (MP2, PBE0, M06, CBSQB3, B3LYP/cc-pVTZ, B3LYP/aug-cc-pVTZ, etc.) were tested. Such an investigation proved that the adopted methods are the best and the most reasonable choice to be compared with the experimental results, insuring a meaningful description of the proposed reaction paths as well. To investigate halogen migration, a detailed analysis of the production of CHClF+, *m*/*z* 67, was conducted, taking into account the transition state energy barrier of the process. All critical points were characterized as energy minima or transition state structures (TS) by calculating the harmonic vibrational frequencies at the same level of theory. They were also used to compute the zero-point and thermal energy corrections [35]. The TS were unambiguously related to their interconnected energy minima by intrinsic reaction coordinate (IRC) calculations [36,37], which are reported in the Supporting Information. Accurate total energies were obtained by single-point coupled cluster calculations, CCSD and CCSD(T) [38], using the same basis sets as for the B3LYP calculations. The CCSD T1 diagnostic is within the recommended threshold of 0.02 [39], suggesting that these species are correctly described by a single-reference method. A detailed description of the theoretical calculation is reported in [40,41].

A natural bond orbital (NBO) analysis [16,42] was conducted to obtain further insights into the nature of the halogen migration as well as the overall reaction mechanisms. The NBO calculations were performed on the available structures through single-point calculations on the optimized structures using DFT with the B3LYP function along with Dunning’s augmented correlation-consistent polarization valence basis set of triple-zeta quality (aug-cc-pVTZ) [17,43,44,45].

These calculations were performed using the NBO 3.1 package [46] available in GAUSSIAN 09 [47]. Through the second-order perturbation theory, the stabilization energy E(2) between the different Lewis-type donor (filled) and non-Lewis-type acceptor (empty) orbitals was evaluated. The resulting E(2) associated with a delocalization for a donor NBO(i) and acceptor NBO(j) is defined as E(2) = ΔE*_ij_*= q*_i_*F(*i*,*j*)^2^/(ε*_j_* − ε*_i_*), where q_i_ is the donor orbital occupancy, ε_i_, and ε_j_ are the orbital energies, and F(i,j) is the off-diagonal NBO Fock matrix element. The outcome of these calculations provides an estimation of the delocalized interactions and can give an indication of the most favorable reaction path.

The outer valence vertical ionization energies (IEs) were calculated using the outer valence Green’s function OVGF/6-311++G** [48,49] and P3/6-311++G** methods [50,51].

## 5. Conclusions

An investigation of the photofragmentation of halothane was conducted using PEPICO experiments at 21.5 eV and PIE measurements of the AEs of several fragments. The experimental AEs were compared with the predicted ones by DFT calculations. The observation of the CHClF^+^ and CHBrF^+^ fragments proves that, following ionization, the migration of a F atom from the CF_3_ moiety to the BrCClH one can occur. The formation of CHClF^+^ was investigated theoretically. Two distinct reaction paths emerged (Figure 3 and Figure 4), mediated by the TS1 and TS2 species (Figure 8). The coupled cluster calculations and NBO analysis show that the reaction involving the TS2, i.e., where the ejection of the Br atom followed by the formation of a transition state in which the F atom migrates to the adjacent C atom, is the favorite one due to the lower energy and a higher stability. Furthermore, the reaction mechanism involving the TS2, can be framed within the class of 1,2 fluorine radical rearrangement [52], as well as the one related to internal halogen exchange [53].

On one hand, the present results provide useful information on the photochemistry of halothane exposed to VUV radiation, which is relevant for environmental concerns, and on the other on the halogen atom transfer (XAT) process, one of the most important and applied processes for the generation of carbon radicals in synthetic chemistry [54].

## Figures and Tables

**Table 1:** Measured and calculated AEs of the parent ion, M^+^, and some fragments.

(*m*/*z*)	AEexp (eV)	AEth (eV) CCSD	AEth (eV) CCSD(T)	Assignment
198	11.05 ± 0.03	10.83	10.77	M^+^
179	14.22 ± 0.27	13.30	13.28	(M-F)^+^
129	11.98 ± 0.07	11.96	11.93	BrClCH^+^
117	11.68 ± 0.08	11.77	11.81	(M-Br)^+^
111	14.57± 0.06			CHBrF^+^
69	14.20 ± 0.25 *	12.93	12.97	CF_3_^+^
67	14.04 ± 0.19	13.94	13.98	CHClF^+^

* In the case of CF_3_^+^ (*m*/*z* 69), the AE_exp_ was obtained by the first onset of the PEPICO experiments while all the others were obtained by the PIE measurements.

**Table 2:** Calculated ionization energies and pole strengths with OVGF and P3 methods with 6-311++G** basis set.

Orbital	Energy (eV) P3/6-311++G**	Pole Strength	Energy (eV) OVGF/6-311++G**	Pole Strength
46a	11.172	0.920	11.127	0.919
45a	11.294	0.920	11.243	0.919
44a	12.135	0.917	12.039	0.914
43a	12.203	0.916	12.094	0.912
42a	14.906	0.913	14.717	0.907
41a	15.329	0.914	14.966	0.903
40a	16.210	0.915	15.765	0.903
39a	16.594	0.927	15.785	0.910
38a	17.174	0.926	16.424	0.910
37a	17.299	0.924	16.590	0.910
36a	18.272	0.927	17.497	0.909
35a	18.331	0.926	17.56	0.908

**Table 3:** Main NBO interactions of halothane at B3LYP/aug-cc-pVTZ level.

Donor NBO(i)	Acceptor NBO(j)	E(2) kcal/mol	E(j)-E(i) a.u.	F(i,j) a.u.
33.LP(2)F2	358.BD*(1)C1-C4	6.69	0.69	0.061
34.LP(3)F2	357.BD*(1)C1-F3	10.21	0.68	0.075
34.LP(3)F2	359.BD*(1)C1-F6	10.55	0.67	0.076
35.LP(1)F3	48.RY*(2)C1	8.18	2.37	0.124
36.LP(2)F3	358.BD*(1)C1-C4	7.55	0.69	0.065
37.LP(3)F3	356.BD*(1)C1-F2	10.71	0.67	0.076
37.LP(3)F3	359.BD*(1)C1-F6	11.23	0.67	0.078
40.LP(3)Br5	361.BD*(1)C4-Cl7	6.40	0.39	0.045
41.LP(1)F6	47.RY*(1)C1	6.61	2.36	0.112
42.LP(2)F6	358.BD*(1)C1-C4	6.69	0.69	0.061
43.LP(3)F6	356.BD*(1)C1-F2	10.36	0.67	0.075
43.LP(3)F6	357.BD*(1)C1-F3	10.54	0.68	0.076
46.LP(3)Cl7	360.BD*(1)C4-Br5	9.13	0.36	0.051

**Table 4:** Main NBO interactions of parent ion (halothane+) at B3LYP/aug-cc-pVTZ level for alpha and beta spin.

**ALPHA SPIN**				
**Donor NBO(i)**	**Acceptor NBO(j)**	**E(2) kcal/mol**	**E(j)-E(i) a.u.**	**F(i,j) a.u.**
34.LP(3)F1	358.BD*(1)C2-F7	6.32	0.68	0.084
34.LP(3)F1	359.BD*(1)C2-F8	5.82	0.69	0.08
43.LP(3)F7	359.BD*(1)C2-F8	6.63	0.69	0.086
45.LP(2)F8	356.BD*(1)F1-C2	7.67	0.68	0.092
**BETA SPIN**				
**Donor NBO(i)**	**Acceptor NBO(j)**	**E(2) kcal/mol**	**E(j)-E(i) a.u.**	**F(i,j) a.u.**
35.LP(3)F1	357.BD*(1)C2-F7	6.35	0.68	0.084
35.LP(3)F1	358.BD*(1)C2-F8	5.78	0.69	0.08
41.LP(2)F7	355.BD*(1)F1-C2	6.18	0.68	0.083
44.LP(2)F8	355.BD*(1)F1-C2	6.57	0.68	0.085
45.LP(3)F8	357.BD*(1)C2-F7	7.92	0.68	0.093

**Table 5:** Main NBO interactions of TS1 at B3LYP/aug-cc-pVTZ level for alpha and beta spin.

**ALPHA SPIN**				
**Donor NBO(i)**	**Acceptor NBO(j)**	**E(2) kcal/mol**	**E(j)-E(i) a.u.**	**F(i,j) a.u.**
5.BD(1)C2-Cl6	361.BD*(2)C2-Cl6	5.57	0.61	0.075
34.LP(3)F3	359.BD*(1)C1-Br7	5.78	0.41	0.063
36.LP(2)F4	357.BD*(1)C1-F3	7.70	0.60	0.086
361.BD*(2)C2-Cl6	360.BD*(1)C2-Cl6	7.49	0.12	0.118
**BETA SPIN**				
**Donor NBO(i)**	**Acceptor NBO(j)**	**E(2) kcal/mol**	**E(j)-E(i) a.u.**	**F(i,j) a.u.**
5.BD(1)C2-Cl6	360.BD*(2)C2-Cl6	5.79	0.60	0.077
35.LP(3)F3	357.BD*(1)C1-F4	5.38	0.61	0.073
35.LP(3)F3	358.BD*(1)C1-Br7	5.04	0.42	0.059
38.LP(3)F4	358.BD*(1)C1-Br7	6.69	0.41	0.067
360.BD*(2)C2-Cl6	359.BD*(1)C2-Cl6	11.59	0.10	0.123

**Table 6:** Main NBO interactions of TS2 at B3LYP/aug-cc-pVTZ level for alpha and beta spin.

**ALPHA SPIN**				
**Donor NBO(i)**	**Acceptor NBO(j)**	**E(2) kcal/mol**	**E(j)-E(i) a.u.**	**F(i,j) a.u.**
4.BD(1)F4-C5	31.LP*(1)C1	34.14	0.37	0.162
34.LP(3)F2	31.LP*(1)C1	29.46	0.25	0.124
37.LP(3)F3	31.LP*(1)C1	29.19	0.25	0.123
40.LP(3)F4	31.LP*(1)C1	20.27	0.19	0.090
40.LP(3)F4	360.BD*(1)F4-C5	5.98	0.33	0.058
43.LP(3)Cl6	360.BD*(1)F4-C5	15.64	0.24	0.079
**BETA SPIN**				
**Donor NBO(i)**	**Acceptor NBO(j)**	**E(2) kcal/mol**	**E(j)-E(i) a.u.**	**F(i,j) a.u.**
4.BD(1)C1-Br7	360.BD*(1)F4-C5	7.89	0.14	0.044
5.BD(1)F4-C5	359.BD*(1)C1-Br7	35.85	0.37	0.161
33.LP(2)F2	357.BD*(1)C1-F3	7.45	0.69	0.091
34.LP(3)F2	359.BD*(1)C1-Br7	26.87	0.26	0.117
36.LP(2)F3	356.BD*(1)C1-F2	7.35	0.69	0.09
37.LP(3)F3	359.BD*(1)C1-Br7	26.52	0.26	0.117
40.LP(3)F4	359.BD*(1)C1-Br7	20.10	0.20	0.089
40.LP(3)F4	360.BD*(1)F4-C5	6.01	0.33	0.059
43.LP(3)Cl6	360.BD*(1)F4-C5	15.02	0.24	0.078

## Data Availability

The data are available on reasonable request from the corresponding author.

## Supplementary Materials

The following supporting information can be downloaded at https://www.mdpi.com/article/10.3390/molecules30142902/s1.
