# Supplementary material for: Halogen Migration in the Photofragmentation of Halothane
Source: Molecules. 2025 Jul 9;30(14):2902. doi: 10.3390/molecules30142902 (PMC12299474; doi:10.3390/molecules30142902)
Supplement: Supplementary file 1 [file molecules-30-02902-s001.zip › molecules-3688692-supplementary.pdf]

Halogen migration in the photofragmentation of Halothane

Anna Rita Casavola[1] , Filippo Morini[2,*], Mattea Carmen Castrovilli[1], Jacopo Chiarinelli[1], Laura Carlini[1], Antonella Cartoni[1,3], Daniele Catone[4], Paola Bolognesi[1], Robert Richter[5], Bratislav Marinkovic[6], Sanja Tosic[6] and Lorenzo Avaldi[1]

Supporting Information

IRC curves for the calculation of the TS , which show that the TS links the correct reactants and products for the two pathways discussed in the main text.

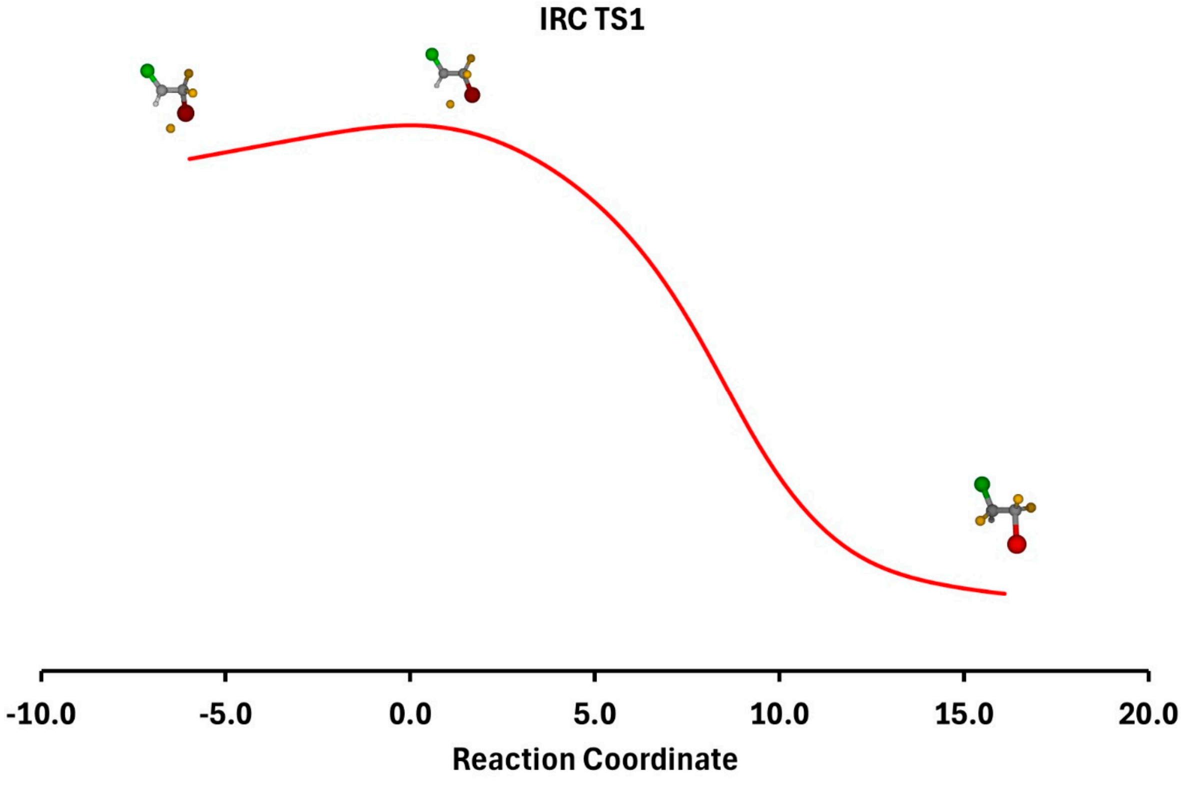

Figure S1. IRC for TS1.

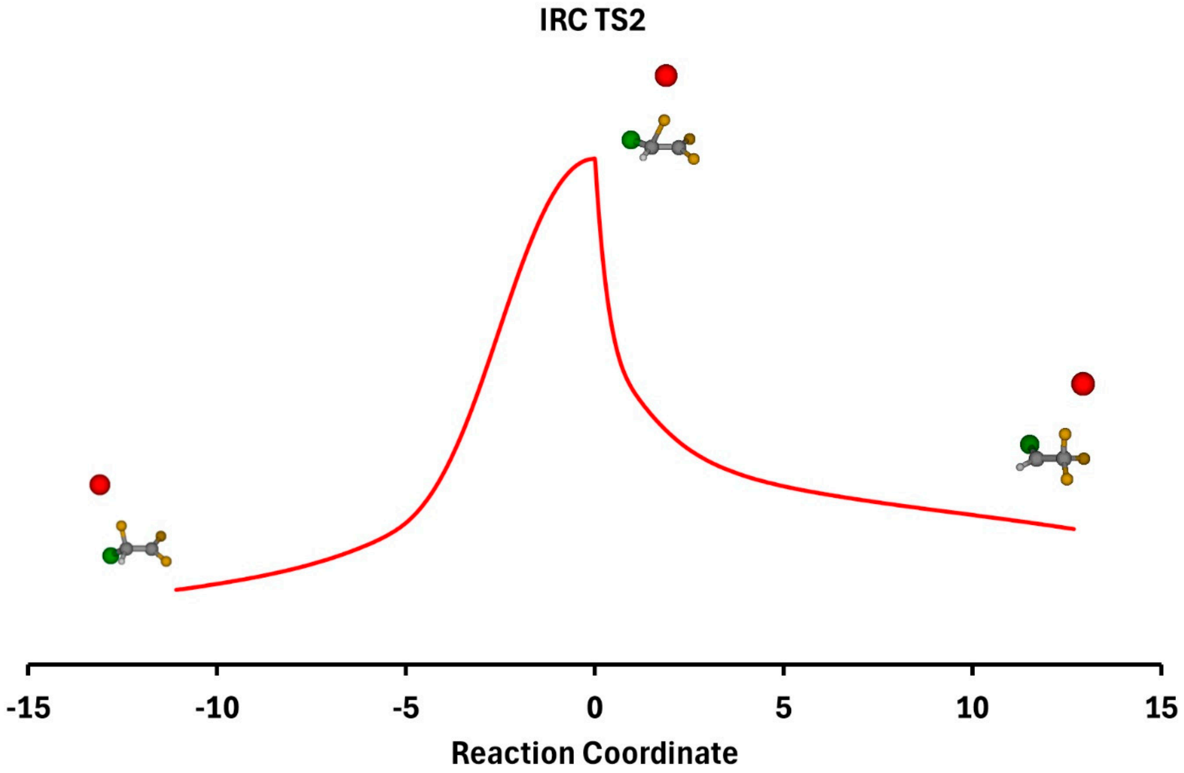

Figure S2. IRC for TS2.

The studied geometries have been optimized by means of Density Functional Theory (DFT) along with the Becke-3-parameters-LeeYang-Parr (B3LYP) functional and the 6-311++g** basis set. All the optimized xyz matrix of the neutral molecule M, the cation M^+ and several fragments are reported in the following tables.

8

C	0.000000	0.000000	0.000000
F	0.000000	0.000000	1.346335
F	1.270408	0.000000	-0.416092
C	-0.802190	1.203197	-0.519771
Br	-0.923502	1.165520	-2.471162
F	-0.577049	-1.147048	-0.400612
Cl	-0.109009	2.727181	0.086974
H	-1.817440	1.122592	-0.144043

Table S1: Optimized structure in xyz format of M

8

C	0.000000	0.000000	0.000000
F	0.000000	0.000000	1.327969
F	1.244773	0.000000	-0.452765
C	-0.779024	1.253260	-0.463968
Br	-0.758151	1.505634	-2.412395
F	-0.647727	-1.068965	-0.456757
Cl	0.040378	2.792544	-0.140690
H	-1.803971	1.268891	-0.108609

Table S2: Optimized structure in xyz format of M^+

7

C	0.000000	0.000000	0.000000
Cl	0.000000	0.000000	1.718322
H	0.989101	0.000000	-0.450743
Br	-1.052748	-1.491391	-0.919122
C	-0.955252	0.868182	-0.688460
F	-2.008081	1.304249	-0.139650
F	-0.682221	1.393853	-1.811454

Table S3: Optimized structure in xyz format of $(M\text{-}F)^+$

4

C	0.000000	0.000000	0.000000
Cl	0.000000	0.000000	1.637135
H	0.968189	0.000000	-0.499476
Br	-1.468104	0.000468	-1.028775

Table S4: Optimized structure in xyz format of $BrClCH^+$

7

C	0.000000	0.000000	0.000000
C	0.000000	0.000000	1.545534
Cl	1.336587	0.000000	2.430906
F	-0.664009	1.093564	-0.356942
F	-0.661068	-1.095433	-0.357445
F	1.215370	0.001994	-0.500171
H	-0.970193	-0.000288	2.049401

Table S5: Optimized structure in xyz format of $(M\text{-}Br)^+$

4

C	0.000000	0.000000	0.000000
F	0.000000	0.000000	1.235619
F	1.070250	0.000000	-0.618023
F	-1.070177	-0.000174	-0.617638

Table S6: Optimized structure in xyz format of CF_3^+

4

C	0.000000	0.000000	0.000000
Cl	0.000000	0.000000	1.617470
H	0.926387	0.000000	-0.581395
F	-1.078736	0.000000	-0.640655

Table S7: Optimized structure in xyz format of $CHClF^+$

8

C	0.519816	-0.158796	0.045288
C	0.079491	-0.247502	1.488019
F	1.779073	-0.680739	-0.112077
F	-0.366727	-0.812843	-0.767795
F	-1.606354	1.275982	0.426754
Cl	0.486226	-1.575281	2.437568
Br	0.529318	1.799347	-0.373922
H	-0.532646	0.538058	1.923756

Table S8: Optimized structure in xyz format of TS1

8

C	0.000000	0.000000	0.000000
C	0.000000	0.000000	1.442887
Cl	1.479135	0.000000	2.351825
F	1.092128	-0.159908	-0.715731
F	-1.070047	0.345492	-0.686550
F	-0.384171	-1.578567	0.850249
Br	-0.974255	-4.168333	0.915215
H	-0.896640	0.313981	1.966442

Table S9: Optimized structure in xyz format of TS2

On the optimized structures, CCSD(T)/6-311++G** energy calculations have been done.
